# Hospital-based, prospective, multicentre surveillance to determine the incidence of intussusception in children aged below 15 years in Germany

**DOI:** 10.1186/1471-230X-11-26

**Published:** 2011-03-24

**Authors:** Nicolai Bissantz, Andreas C Jenke, Matthias Trampisch, Renate Klaaßen-Mielke, Kathrin Bissantz, Hans-Joachim Trampisch, Tim Holland-Letz

**Affiliations:** 1 University of Bochum, Fakultät für Mathematik, D-44780 Bochum, Germany; 2 HELIOS Children´s Hospital, Witten/Herdecke University, Heusnerstr. 40, D-42283 Wuppertal, Germany; 3 University of Bochum, Department of Medical Informatics, Statistics and Epidemiology, D-44780 Bochum, Germany; 4 Schattbachstrasse, 44801 Bochum, Germany

## Abstract

**Background:**

A new vaccine against Rotavirus (RV) gastroenteritis was introduced in Germany in 2006. In 1997 the first RV vaccine was withdrawn due to an increased incidence in intussusception (IS). Thus, an accurate estimation of the incidence of IS is important for post-licensure surveillance.

**Methods:**

IS-Data were obtained from the 'Erhebungseinheit für seltene pädiatrische Erkrankungen Deutschland' (ESPED, German surveillance unit for rare pediatric diseases) collaborations' central register where all cases of intussusception in Germany for the years 2006 and 2007 are collected (n = 1200). In order to obtain an unbiased estimate of the incidence, it is necessary to determine the population under risk out of which these cases originated, and the proportion of real cases not reported to the registry (underreporting). In order to assess underreporting, a random sample of 31 hospitals was re-assessed by an outside reviewer. The estimation of incidence was done using a single Maximum-Likelihood (ML) estimator based on data from both the registry and the sample.

**Results:**

The uncorrected observed incidence was calculated to be 26.6/100,000 child-years for children below 1 year old, 23.8 for those below 2 years old, and 5.2 for those below 15 years old. The review revealed a mean reporting quota of about 41% and the ML approach yielded an incidence of 51.5/100,000 child-years (95%CI [41.7;61.1]) for children below 2 years of age.

**Conclusions:**

While substantial under-reporting led to very conservative estimates of the IS incidence, the approach described here allows an accurate estimation of IS incidence including corresponding confidence bands. Therefore, ML estimation is a straightforward instrument to derive stable, unbiased estimates in epidemiological studies with incomplete data.

## Background

Intussusception (IS) is characterized by the inversion of an intestinal section into an adjacent intestinal section [1]. Main symptoms are abdominal pain, abdominal resistance and bloody tinged defecation. IS leads to obstruction of bowel passage and the venous blood flow, resulting in a sudden onset of colicky abdominal pain, and constriction of the mesentery [2]. Classification of IS occurs by demonstration of intestinal invagination via surgery, radiology, and/or autopsy. Incidence data on IS show a wide variation, ranging from 35 (Brazil) to 1200/100,000 child-years (United Kingdom) for children below 1 year [3] which is mainly due to the lack of a standardized case definition. Only recently diagnostic guidelines were defined by the Brighton Collaboration Working Group [1].

The importance of accurate incidence estimation on the base of standardized diagnostic criteria is even more important on the background of vaccination against Rotavirus (RV) gastroenteritis. This is because the first vaccine against RV, a tetravalent, rhesus-based rotavirus vaccine (RRV-TV), approved by the United States Food and Drug Administration in 1998, was withdrawn from the market following reports of IS among recently vaccinated children [2]. Nevertheless, more than 600,000 infants received at least one dose [4]. At that time the risk of IS related to the vaccine has been estimated to be anywhere between 1 in 4,670 and 1 in 32,000 vaccinated children [2,5,6].

Just recently, two new RV vaccines, Rotarix© and RotaTeq©, have been introduced on the market. To provide a clear epidemiological statement about IS following vaccination, the incidence of IS has to be determined prior to global introduction of these new vaccines in order to establish baseline disease incidence rates and allow for interpretation of post-licensure IS surveillance data [7].

Between January 1, 2006, and December 31, 2007, a nationwide, active hospital based, multicenter surveillance study was conducted by ESPED to determine the incidence of IS in Germany. Even though more than 99% of all German pediatric hospitals participated, one challenge in this study and for surveillance studies in general, was potential incomplete reporting of IS cases. This might result in an underestimation of the incidence.

A similar but much smaller study was recently performed in Switzerland. Uncorrected IS incidence obtained from this surveillance was refined by means of a Capture-Recapture analysis [8]. After correction, the incidence of IS was estimated to be 56/100,000 child-years in children below 1 year compared to 38/100,000 initially and 46/100,000 for children below 2 years of age compared to 31/100,000 initially.

We expected similar under-reporting in Germany, so we decided to estimate the fraction of reported cases by a thorough review of a 10% random sample of the participating hospitals. It was assumed that the distribution of under-reporting in the random sample corresponds to the distribution of under-reporting in the annual incidence estimated through the ESPED surveillance. Thereby it was possible to calculate adjusted incidence estimates for a given age group.

Since it was necessary to include several probabilistic steps in the generation of the data, the necessary model is rather complex. The model has to include the true (random) number of cases in a hospital's population under risk, and must also allow randomness of the reporting quota of a hospital. This reporting quota is not identical for all hospitals, but can be modeled as a random realization based on the binomially distributed number of reported cases (using the true number of cases and the true reporting quote of the hospital as parameters). Two groups of clinics have to be distinguished according to the availability of the true number of IS cases, i.e. those hospitals included in the sample where the true reporting quota is available and the hospitals not in the sample where this is not the case. It is not possible to fit this rather complicated data model into a standard method. Instead, a more sophisticated statistical analysis is required which is provided by maximum likelihood estimation. This method is commonly used for complicated data structures such as phenotype analysis in genetic applications (cf. e.g. [9,10]). To our knowledge, it has never been used so far for estimation of clinical incidence. The basic idea of this method is to compute the probability of observing data for all possible values of the parameter which is to be estimated (i.e. the incidence α). In this paper we describe our method for the estimation of α, followed by an explanation of the construction of the associated confidence bands by bootstrap methods. Finally, we use the method to determine estimates of the incidence and their associated confidence intervals.

## Methods

### ESPED study period and study design

This study was a prospective, hospital-based, multicenter surveillance study conducted from January 1, 2006 to December 31, 2007. The study protocol was approved by the institutional review board and the ethics committee of Witten/Herdecke university and also approved by the data protection commission of the state of North Rhine Westfalia.

### Study population

The target population for this study were children under 15 years of age with a diagnosis of IS reported to ESPED by participating pediatric medical care providers during the study. However, the aim is to obtain an estimate for the incidence of IS valid for all children below 15 years in Germany.

### Inclusion criteria

IS cases were included if the defined eligibility criteria given below were met:

• male or a female child below 15 years of age at the time of enrolment into the study

• subject fulfilling Brighton Collaboration's Group Criteria (version dated January 30,2002) for definite IS [1]

• IS diagnosis occurring during the two years study period.

### Exclusion criteria

Patients were not included as IS cases if the criteria below were met:

• diagnosis of IS based solely on clinical signs and symptoms

• means for diagnosis of IS cannot be determined.

### ESPED data collection

#### The ESPED reporting system

Data were collected by ESPED, which performs active surveillance of rare pediatric conditions in Germany based on monthly contact to pediatric care providers. More than 99% of the pediatric departments in Germany (including departments for pediatric surgery) are members of ESPED for at least one of the medical conditions under observation. The ESPED system has just recently been reviewed and was found to have generated more than 100 publications between January 1992 and August 2008. Seven of those publications had an impact factor above 10. Generally, more than 90% of all German pediatric hospitals participated in surveillance studies if the principal investigator was supported by staff comprising at least two persons or if the mailing of the questionnaire was handled by the ESPED office - as in this study [11].

**Figure 1:**
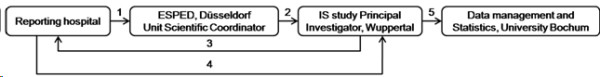
**Structure of the ESPED reporting system (IS reporting)**.

The participating hospitals complete an internet based form with a list of conditions under observation and send this back to ESPED electronically (see Figure 1).

Reports received by the Unit's Scientific Coordinator at ESPED headquarters are forwarded to appropriate investigators depending on the reported condition.

For this study, trial reports on IS were forwarded to the IS study Principal Investigator in Wuppertal, who contacted the reporting clinicians directly for completion of a detailed follow-up questionnaire. These follow-up questionnaires were directly returned to the IS study principal investigator. To ensure return of all completed follow-up questionnaires, all hospitals with at least three missing questionnaires were personally visited by a medical student.

To get an unbiased estimation of the distribution of under-reporting, a random sample of 46 out of the 336 participating hospitals was asked to review all cases of IS between Jan. 1, 2006 and Dec. 31, 2007. Thirty-one hospitals agreed to allow review of their data. The review was done by a trained medical student who reviewed the ICD-10 and OPS codes in the clinic register and did a retrospective assessment of all eligible patients based on the medical record for fulfilling the Brighton Collaboration Group Criteria for IS diagnosis (level 1). For each identified case of IS, the following information was submitted: gender, date of admission, birth month and year. Based on these variables the reviewed cases were compared with the cases which had been originally reported to ESPED surveillance for each hospital. Thus, an estimate of the underreporting rate for each hospital in the sample is possible. With the distribution of under-reporting rates in the sample and the original ESPED surveillance annual incidence, it is possible to calculate adjusted estimates for a given age group.

### Statistical analysis

#### Estimation of the corrected IS incidence

An uncorrected estimation of the incidence as the ratio between the annual number of IS cases reported through the ESPED network and the number of children at risk (provided by the "Statistisches Bundesamt, the German Federal Office for Statistics, for 2006" [12]) might lead to under-estimation of IS incidence due to under-reporting of cases. Therefore, we used a maximum likelihood approach for the estimation of the corrected incidence taking under-reporting into account.

##### 1. Determination of the population under risk

To determine the population under risk, i.e. the number of children of the corresponding age group within the catchment area of a given hospital, five different models were developed with the aim of modelling the probability that a specific hospital is visited in case of IS. To this end we used administrative district population data for children below 15 years of age obtained from the 'Statistisches Bundesamt' [12]. The following models were used:

• **v0**: The population under risk corresponds to the fraction of pediatric beds of a hospital relative to the overall number of such beds in Germany [13] based on the 'Krankenhausbedarfsplanung' (a forecast on the need of hospitals and pediatric beds). The number of beds was calculated as sum of pediatric and pediatric surgery beds; pediatric cardiology and neonatology beds were not included.

The models **v1 **to **v4 **include the distance to a hospital as a potential factor influencing the choice of the hospital, and its degree of specialization. The distance *r_ij _*of hospital *i *from the centre of a district *j *was determined from GPS coordinates, where hospitals more than 50 km away from a district were not considered. The second influencing factor included was the degree of specialization as a measure of a hospital's quality for IS treatment. Hospitals were labelled type 1, 2 and 3 if they had ≤400, 400 - 800, or >800 beds, respectively, with pediatric hospitals always being of type 3.

• **v1: **The fraction of an administrative areas' population choosing a specific hospital is inversely proportional to the distance, with an additional weighting based on its specialization (weight factor 2/3, 3/3, 4/3 for type 1/2/3 respectively).

• **v2: **Similar to v1, but using the squared distance instead.

• **v3: **As v1, but using third power of distance.

• **v4: **Similar to v2 but using modified weights (8/9; 1; 10/9), thus reducing the impact of specialization.

##### 2. Maximum likelihood estimation of the global incidence α

We use the following notation:

*B_i _*: population within the catchment area of hospital i for a fixed population model v_j_, j = 0,...,4

*K_i _*: number of "all" cases within the catchment area of hospital i (true number)

*M_i _*: number of reported cases of hospital i

*p_i _*: "reporting quota" of hospital i (if available)

*α*: global incidence of IS

The random model for the data is as follows. The true number of IS cases for a hospital Ki is Poisson distributed with parameter α. Bi, i.e.

**Figure 2:**
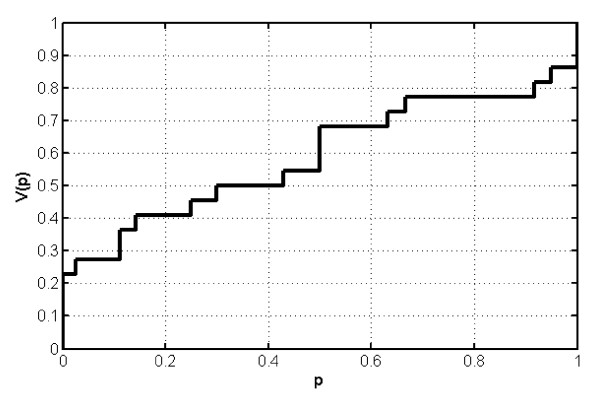
**Empirical distribution function V(p) of the reporting quota p for the age group below 15 years of age**.

For hospitals for which the true number of cases is unknown, the reported number *M*_*i *_is binomially distributed with *K*_*i *_attempts and probability *p*_*i*_:

The reporting quota *p*_*i *_are assumed to be independent and identically distributed between hospitals, i.e. *p_i_~V(p) *with density function *v(p)*, for all hospitals, and also independent of *K*_*i*_. Refer to Figure 2 for a plot of the empirical distribution function *V(P) *of the reporting quota *p*.

The maximum likelihood estimator for the incidence is based on the likelihood function L(*α)*, i.e. the probability to observe the data given the respective value of the incidence *α*. Hospitals in the sample with *K_i _*available are treated separately from hospitals for which only *M_i _*is known. Consequently, we are concerned with the product of two separate likelihood functions L_1_(α) and L_2_(α).

The likelihood *L_1 _*for hospitals in the sample with known *M*_*i *_and *K*_*i *_is

For the second group of hospitals only the number of reported IS cases M_i _is available. To deal with the lack of knowledge about the actual distribution of under-reporting of IS cases *V(p) *we estimate it from the observed under-reporting rates in the first group of hospitals. This yields

**Figure 3:**
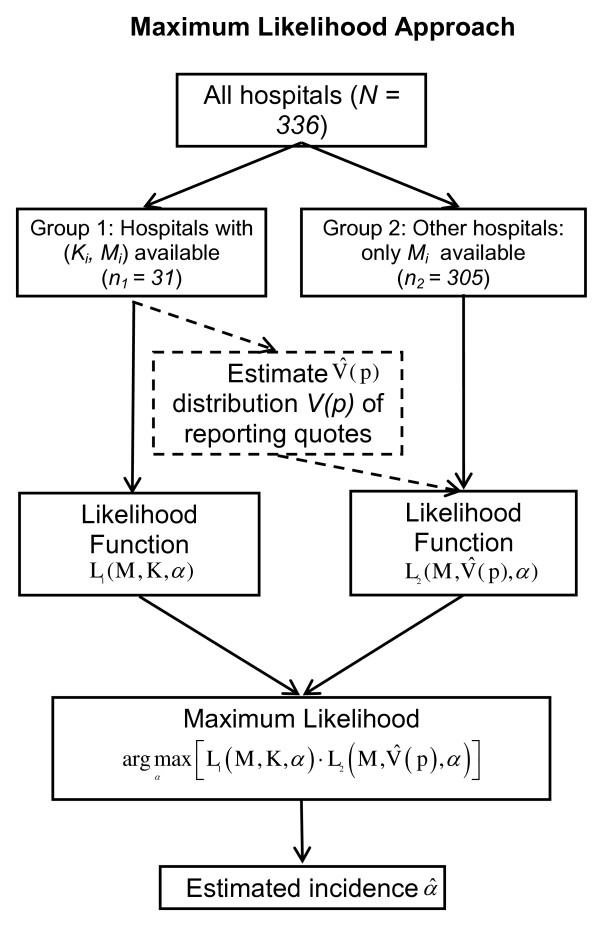
**The maximum likelihood method**.

The likelihood of the full dataset is L(α) = L_1_(α)·L_2_(α) (cf. Figure 3). Maximizing this term with regard to α will yield the maximum likelihood estimate for the true incidence α.

##### 3. Determination of confidence bounds for the global incidence α

**Figure 4:**
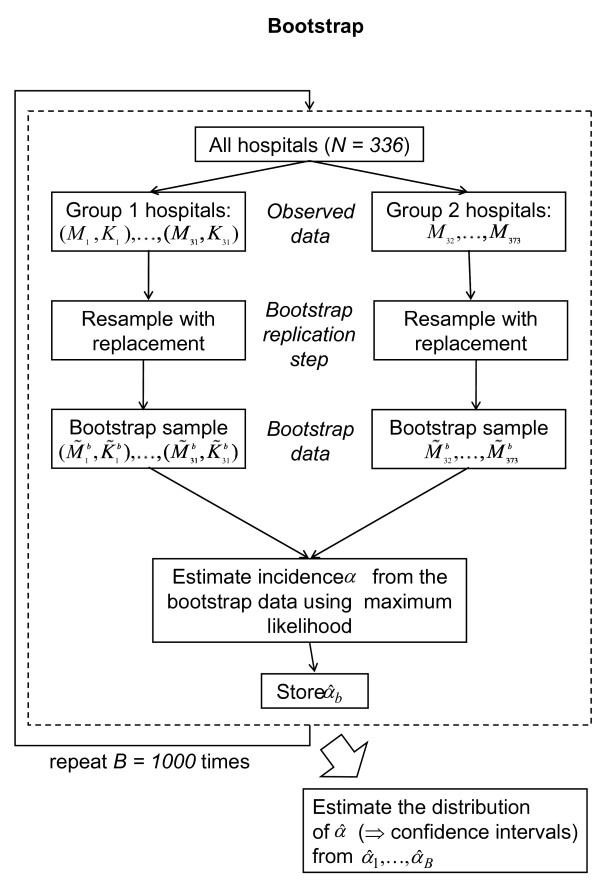
**The bootstrap method**.

Having obtained an estimate for the incidence, we still need a confidence bounds as an assessment for the precision of the estimate. Confidence bounds are obtained with a bootstrap approach (see Figure 4[14]). It consists of executing *B *times the following steps, yielding a sample of bootstrap incidences :

1. Random selection of a population model from v0,..., v4 (with equal chance for each choice).

2. Generation of a bootstrap data sample from the original data (M_1_, K_1_),...,(M_n1_, K_n1_), and M_n1+1_,..., M_n2 _by sampling with replacement n_1 _and n_2 _observations from (M_1_, K_1_),...,(M_n1_, K_n1_) and M_n1+1_,..., M_n2_, respectively. Hence, the numbers of hospitals with K_i _available (or not) will be the same as in the original data set.

3. Estimation of the incidence α from the bootstrap data using the population model selected in the first step. Performing step 1-3 for the k-th time yields the bootstrap estimate .

The distribution of  closely resembles the true distribution of  (for asymptotical convergence results in similar cases see [14]). Hence, confidence bounds on α can be determined from the quantiles of the bootstrap distribution of , e.g. two-sided confidence bounds with nominal error probability β will be:

Here,  is the estimated IS incidence computed from the original data and . _([x]) _the x-th ordered sample of the  bootstrap estimates. For our analysis we use B = 1,000.

## Results

### Study population

Currently, there are 373 pediatric departments (including departments for pediatric surgery) reporting data to ESPED on any medical condition under observation. Out of these 373 departments, 91 to 94% send monthly reports back to ESPED headquarters, and 336 hospitals were eligible to be included in the estimation of IS incidence. Forty-seven hospitals were excluded because they were not expected to treat IS cases due to their specialization as for example pediatric cardiac surgery units.

With regard to IS surveillance, 241 of the 336 departments reported cases of IS (71.7%). 209 of the departments with one or more reported cases sent back at least one completed follow-up questionnaire (86.7%).

**Figure 5:**
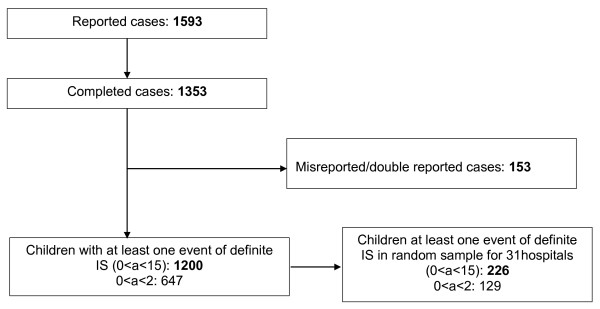
**Study population**.

From January 1, 2006 to December 31, 2007, 1,593 cases with IS were reported through the internet-based reporting form (see Figure 5). For 1,353 reported IS cases (84.9%) the follow-up questionnaires were returned (i.e. cases referred to as completed cases). A total of 153 cases (11.3% of the completed cases) were either double reports or misreported as IS, and were therefore excluded from the final analyses. Consequently, the analysis was performed with 1,200 children with at least one definite (according to the case definition criteria) occurrence of IS.

### Calculation of the empirical distribution function of underreporting

To get an unbiased estimation of the fraction of under-reporting, a random sample of 46 hospitals out of the 336 hospitals was asked to re-evaluate all IS cases occurred in the observation period from their records. Thirty-one hospitals agreed to review their data. For these 31 hospitals the number of pediatric beds and the degree of specialization were compared with the 305 remaining hospitals, which yielded no statistically significant difference.

For the age group below 15 years of age, 9 of the 31 hospitals did not report any IS case to ESPED nor revealed any case during the re-evaluation; therefore, they did not contribute to the empirical distribution of the reporting quota. This is the case for 10 hospitals for the age group below two years of age and for 11 hospitals for the age group below one year of age. A basic assumption of the model is that it is possible to transfer the empirical distribution of the reporting quotes of the random sample to all hospitals. We further assume that the reporting quote is randomly distributed over the hospitals and is independent of characteristics of the hospital. This seems to be justified as there is no significant or relevant correlation between hospital specialization and the reporting quota in all age groups (Spearman's correlation coefficient is 0.181 (p = 0.430) for children below 15 years, 0.137, (p = 0.554) for children below 2 years, and 0.136 (p = 0.569) for children below 1 year.).

The mean reporting quota was 41.1% for age below 15 years closely similar as for age below 2 years, where it was 40.6%. For children aged below 1 year, it was 45.7%.

### Annualized incidence of IS in Germany

An uncorrected annual incidence of IS in Germany for a given age group was calculated as the ratio between the annual number of IS cases reported via the ESPED network and the number of children at risk, i.e. the number of German children within the corresponding age-group. This uncorrected incidence for IS in Germany is α = 5.2/100,000 child-years for children below 15 years, 23.8/100,000 child-years for children below 2 years and 26.6/100,000 child-years for children below 1 year (see Table 1).

**Table 1:** Annualized incidence of IS in Germany

					Definite IS rate per 100,000 child years	
					
Age (a) at diagnosis (year)	Number of definite completed IS cases	Estimated IS cases based on global incidence**	Time at risk years	Population at risk*	Uncorrected incidence based reported cases (definite) via ESPED	Corrected incidence ** [95%-CI]
0 < a < 1 year	358	813	2	673,139	26.6	60.4 [48.3;72.1]

0 < a < 2 years	647	1,402	2	1,361,627	23.8	51.5 [41.7;61.1]

All (< 15 years)	1,200	2,371	2	11,441,367	5.2	10.4 [8.6;12.1]

*Population data for 2006 provided by the German Federal Office for Statistics

**The corrected incidence is estimated by a maximum likelihood approach (taking into account the underreporting)

Uncorrected annual IS incidence α in Germany based on the ESPED data and corrected IS incidence estimated by a maximum likelihood approach which takes into account the underreporting.

Thus, a crude adjustment of the incidence for children aged below 2 year using the underreporting quotas reported above would yield 58.6% annual incidence of IS. However, we are interested in a more precise estimate which also includes confidence bounds. The maximum likelihood estimate which incorporates underreporting yields a mean value for α over all five population models v0 to v4 of 10.4/100,000 child-years (95%CI: [8.6;12.1] (see Table 2), for children of below 2 years of 51.5 (95%CI [41.7;61.1], and for children below 1 year 60.4/100,000 (95%CI [48.3;72.1]).

**Table 2:** Maximum likelihood incidence α of IS in Germany

Population model	Maximum likelihood incidence α per 100,000 child-years; 95% CI		
	**0<a < 1 years**	**0<a < 2 years**	**All**

V0	60.2 [47.9;71.7]	51.5 [41.7;61.1]	10.7 [9.2;12.8]

V1	61.0 [49.5;73.3]	52.1 [42.9;62.3]	10.4 [8.6;12.2]

V2	60.2 [47.9;71.7]	51.2 [41.1;60.5]	10.2 [8.2;11.8]

V3	59.6 [46.7;70.5]	50.8 [40.3;59.7]	10.2 [8.2;11.8]

V4	61.0 [49.5;73.3]	51.9 [42.5;61.9]	10.3 [8.4;12.0]

**Mean value**	60.4 [48.3;72.1]	51.5 [41.7;61.1]	10.4 [8.6;12.1]

Maximum likelihood incidence α of IS in Germany and 95% confidence bounds for the population models v0, v1, v2, v3 and v4

The corrected incidence α and the corresponding 95% confidence bands for the different population models v0, v1, v2, v3 and v4 are very similar (see Table 2).

## Discussion

Rotavirus vaccines were introduced in Germany 2006. In order to evaluate whether or not these vaccines have an impact on the risk of IS, it is essential to have accurate baseline incidence estimates for IS in Germany.

Up to now, there are very few studies which used the Brighton Collaboration's case definition (first draft 2001 [1]). This study tries to fill this gap.

One retrospective study was performed in Singapore [15] analyzing the IS incidence between 1997 and 2004. While the authors report an average incidence of about 60/100,000 child-years for the first year of life over the whole period, it seems that the incidence is declining after the official introduction of the Brighton criteria from about 82 for 1997-2000 to about 34.6 for 2001-2004. Justice et al. [16] - using a similar retrospective study design - report a similar decline in IS incidence for Australia between 1994 and 2000 fairly comparable to the incidence data in Singapore between 1997 and 2000. Again, this seems to be reasonably explained by the introduction of new case definitions during this period. The transferability of the results of the two discussed studies is possibly limited because other studies indicate different IS rates depending on the ethnic background. For example, Webby et al. [17] present significantly different results for Aboriginal and non-Aboriginal children in the Northern Territory of Australia.

A limitation of the described studies - and also of surveillance studies in general - is the potentially incomplete reporting of IS cases which may result in an underestimation of the incidence.

So far, the only prospective study on IS considering underreporting was performed in Switzerland [8]. In that study the uncorrected IS incidence was refined by means of a capture-recapture analysis. After correction, the incidence of IS was estimated to be 56/100, 000 for children below 1 year compared to 38/100,000 initially and 46/100,000 for children below 2 years of age compared to 31 initially. It is worth noting that the method used in [8] does not reflect a truly independent capture-recapture approach in the classical sense as selections on the re-evaluated hospitals and thus restrictions on the population under risk were made. A truly independent capture-recapture approach would need to assess the whole population under risk which was not the case. This may result in a bias of the estimated reporting quote. Another rather strict assumption was that the estimated reporting quote was modeled to be constant and equal for all participating hospitals in order to correct the incidence estimate for IS. This seems unrealistic.

To avoid the practical problems associated with a truly independent capture-recapture approach and to make use of all available information, we estimated the distribution of the reporting quota from a 10% random sample of all participating hospitals. The data from 31 hospitals was thus re-evaluated based on the ICD codes and the Brighton criteria. The mean reporting quota was 41.1% for children below 15 years (40.6% for children below 2 years and 45.7% for children below 1 year), meaning less than half of the IS-cases were actually reported. For the Swiss Pediatric Surveillance Unit data [8], the reporting quote was estimated to be about 68%.

For our modelling approach the empirical distribution of the reporting quota was an essential part. We had only 5 cases (2%) reported to ESPED which could not be matched definitely to cases in the random sample, which supports the assumption that the review procedure is able to detect very close to 100% of all existing cases of IS.

Another critical model assumption is the independence of the reporting quota and the properties of a hospital: our data set does not provide any evidence of a violation of this assumption. Yet, a possible weakness of our analysis could be that from the selected 41 hospitals only 31 (67.4%) participated in the random sample. However, this sample still seems to represent the remaining hospitals in their known properties very well.

A strong point of the present study was the relatively large number of 1200 cases, which provides a much more solid database than, for example, the 288 cases of the Swiss study. In addition, the maximum likelihood approach used to estimate the corrected IS incidence α takes into account the empirical distribution of the reporting quota. The derived confidence intervals also reflect the actual situation more accurately than just a point estimate.

Our corrected incidence estimated here is 60.4/100,000 child-years for children below 1 year, 51.5/100,000 child-years for children below 2 years of age, and 10.4/100,000 child-years for children below 15 years. These numbers are a little higher than in the Swiss study, yet of the same magnitude.

## Conclusion

In summary, the present study provides a stable and accurate estimation of IS incidence in Germany, corrected for under-reporting. The robustness and accuracy of the used ML model is demonstrated, on one hand, by the very similar values of the corrected incidence α using either one of the five population-under-risk models, and - on the other hand - by the narrow confidence bounds for α obtained from the bootstrap approach.

## Competing interests

The authors declare that they have no competing interests.

## Authors' contributions

All authors were involved in drafting and revising the manuscript. The additional individual contributions were as follows: NB developed the statistical approach. ACJ was project leader clinical part of the IS surveillance. MT contributed to the statistical model. RKM organized the data base and contributed to the statistical analysis. KB contributed to the presentation of the data and did the initial draft of the manuscript. HJT proposed using ML-estimation, obtained funding and participated in the coordination of the study. THL is the overall project supervisor and participated in the conception and design of the study. Moreover, all authors read and approved the final manuscript.

## Pre-publication history

The pre-publication history for this paper can be accessed here:

http://www.biomedcentral.com/1471-230X/11/26/prepub
